# Is symmetric dimethylarginine superior to creatinine for assessing glomerular filtration rate for cats with kidney disease?

**DOI:** 10.18849/ve.v8i4.661

**Published:** 2023-10-04

**Authors:** Lorna Hardy+

**Keywords:** SYMMETRIC DIMETHYLARGININE, SDMA, CREATININE, CKD, CHRONIC KIDNEY DISEASE, GLOMERULAR FILTRATION RATE, GFR, FELINE, CATS

## Abstract

**PICO question:**

Is symmetric dimethylarginine (SDMA) superior to creatinine for assessing glomerular filtration rate (GFR) for cats with chronic kidney disease?

**Clinical bottom line:**

**Category of research:**

Diagnosis.

**Number and type of study designs reviewed:**

The literature searches returned four relevant papers that answered this PICO. Three of the studies were retrospective diagnostic test studies, and one was a randomised, controlled, unblinded study.

**Strength of evidence:**

Moderate.

**Outcomes reported:**

Three out of the four studies analysed found that there was no significant difference between the correlation between symmetric dimethylarginine (SDMA) and glomerular filtration rate (GFR) and creatinine (sCr) and GFR, whilst the other identified a stronger correlation between SDMA and GFR. Two papers also evaluated the sensitivity and specificity of SDMA compared to creatinine. One paper found a similar sensitivity for both biomarkers and a significantly higher specificity for creatinine using the upper reference interval of 18 µg/dL for SDMA and 155.6 µmol/L (1.76 mg/dL) sCr, whilst the other found SDMA to have a superior sensitivity and both biomarkers to have a similar specificity using the upper reference interval of 14 µg/dL for SDMA and 185.64 µmol/L (2.1 mg/dL) for sCr.

**Conclusion:**

In view of the strength of evidence we conclude that the majority of the studies analysed do not demonstrate that SDMA is superior to creatinine for the assessment of GFR in cats with chronic kidney disease. More research is needed with larger sample sizes to investigate this further. Standardisation of the upper reference intervals across studies for creatinine and SDMA would be advantageous for comparison of sensitivity and specificity in future studies.

## How to apply this evidence in practice

The application of evidence into practice should take into account multiple factors, not limited to: individual clinical expertise, patient’s circumstances and owners’ values, country, location or clinic where you work, the individual case in front of you, the availability of therapies and resources.

Knowledge Summaries are a resource to help reinforce or inform decision making. They do not override the responsibility or judgement of the practitioner to do what is best for the animal in their care.

## Clinical scenario

You are performing a senior health check on a cat and are performing bloodwork to assess for chronic kidney disease. Your results have discordant symmetric dimethylarginine (SDMA) and creatinine values that would place the cat in different stages according to the International Renal Interest Society (IRIS) guidelines and you would like to know which result better correlates to kidney function.

## The evidence

The literature searches on Pubmed and CAB Abstracts returned four relevant papers that answered this PICO. Three of the studies were retrospective diagnostic test studies (Hall et al., 2014a; Braff et al., 2014; and Bran et al., 2021), and one was a cross sectional study from a randomised control trial (Hall et al. 2014b). All four studies analysed the correlation between symmetric dimethylarginine (SDMA) and glomerular filtration rate (GFR) and creatinine and GFR. Two of the papers also examined the sensitivity and specificity of SDMA and creatinine for the detection of reduced GFR (Brans et al., 2021; and Hall et al., 2014a). Critical appraisal of the selected papers collectively provide moderate to strong evidence in terms of their experimental design and implementation. Overall, the majority of the studies did not demonstrate that SDMA is superior to creatinine for assessment of GFR in cats with chronic kidney disease.

## Summary of the evidence

**Brans et al. (2021):** 

Population:	Adult, privately owned cats with chronic kidney disease (CKD), diabetes mellitus (DM), or healthy cats that had undergone glomerular filtration rate (GFR) estimation and general health screening as part of previously published prospective studies. Exclusion criteria: Those with insufficient plasma samples for symmetric dimethylarginine (SDMA) measurement. Unknown serum creatinine (sCr) or GFR. Hyperthyroid cats (thyroxine may alter SDMA independent of GFR).
Sample size:	49 cats: 17 cats with CKD – determined by a sCr of > 161.8 µmol/L in combination with urine specific gravity (USG) < 1.035 with relevant clinical exam findings and history. 15 cats with diabetes mellitus (DM). 17 healthy control cats.
Intervention details:	sCr and GFR data were retrieved from the medical files of the cats GFR had been measured using a plasma exogenous iohexol clearance test. GFR was used as a direct measure of filtration function. SDMA was measured from residual clearance test plasma samples that had been stored between 3–8 years prior to SDMA testing. The sensitivity and specificity of the indirect renal markers, SDMA and sCr, were set for two different GFR cut-off values: 1.7 ml/(min kg) = mildly impaired renal function. 1.2 ml/(min kg) = significantly impaired renal function consistent with CKD. Cut-off values for SDMA and sCr to detect reduced GFR were set at two different thresholds. SDMA: 14 μg/dL and 18 µg/dL. sCr: 155.6 μmol/L (1.76 mg/dL) and 161.8 μmol/L (1.83 mg/dL).
Study design:	Retrospective diagnostic test study.
Outcome studied:	Strength of the relationship between SDMA and GFR and between sCr and GFR.Sensitivity, specificity and 95% confidence intervals of SDMA and sCr at the two different cut-off values for each and at two different levels of GFR impairment.
Main Findings (relevant to PICO question)	There was an equally significant (P <0.001) moderate correlation between plasma SDMA and GFR (*τ* *_B_* = −0.57), between sCr and GFR (*τ* *_B_* = −0.56), and between SDMA and sCr (*τ* *_B_* = 0.52). High specificity (96.4% for SDMA and 92.9% for sCr) was found when identifying cats with a GFR < 1.7 ml/(min kg), using a SDMA cut-off of 18 µg/dL and sCr threshold of 155.6 µmol/L (1.76 mg/dL). Sensitivity was lower at 71.4% for SDMA and 76.2 for sCr. Eight cats (5/49 with DM, 3/49 healthy) had normal sCr but increased SDMA, six of which had normal GFR. Two (2/17) cats categorised as CKD had plasma SDMA within reference interval and increased sCr. One demonstrated mild reduction in renal clearance and the other severe. GFR values indicated that the majority of cats with conflictingly high SDMA and normal sCr were false positive SDMA results. Using the upper limit of SDMA and sCr values, SDMA suspected more healthy cats with normal GFR as having impaired renal function (n = 7) compared to creatinine (n = 1). SDMA offered little added diagnostic value compared to sCr. Diagnostic performance of both markers improved as renal impairment progressed. 18 µg/dL is suggested as the upper reference interval for SDMA that does not generate as many false positives.
Limitations:	To be included a residual blood sample (from a previous study) was required which meant that cases were not randomly selected. There were only a small number of cats representing mild renal impairment CKD, therefore limited evaluation in this subset. Some of the samples were stored for up to 8 years, long-term stability for SDMA has not been determined. Allocation into subgroups were based on physical exam and routine laboratory work, however three classified as ‘healthy’ and two DM cats had impaired GFR. SDMA was quantified using the SDMA IDEXX test rather than liquid chromatology mass spectrometry test.

**Braff et al. (2014):** 

Population:	Client owned cats selected from a population of 89 cats in which glomerular filtration rate (GFR) had been previously measured in another study.
Sample size:	10 cats: Four cats had creatinine concentrations above the laboratory reference range (upper limit defined as >2 mg/dL). Six cats had creatinine concentrations within the reference range. GFR ranged from 0.54–2.37 ml/min/kg.
Intervention details:	Creatinine values obtained from previous study data. GFR determined by plasma iohexol clearance in previous study. GFR ranged from 0.54–2.37 ml/min/kg. Symmetric dimethylarginine (SDMA) was calculated using high performance liquid chromatography (HPLC). Frozen samples from the previous study were used.
Study design:	Retrospective diagnostic test study.
Outcome studied:	Relationship between SDMA and GFR, creatinine and GFR, and SDMA and creatinine.Comparison of the correlation between SDMA and GFR, creatinine and GFR, and between the two biomarkers.
Main Findings (relevant to PICO question)	A significant linear relationship (P <0.001) was found between both SDMA and GFR (*R* ^2^ = 0.82) and creatinine and GFR (*R* ^2^ = 0.81). Therefore, they determined that SDMA and creatinine performed equally in detecting changes in GFR. A linear relationship was found between SDMA and serum creatinine (*R* *^2^* = 0.73, P = 0.0017).
Limitations:	There was selection bias in that cats were selected from the previous dataset to provide a range of GFR. Small population studied. The length of time of sample storage was not defined and the preservation of SDMA in stored samples has not been determined.

**Hall et al. (2014a):** 

Population:	Cats selected from a colony of over 400 domestic shorthair cats ranging in age from 1–19 years of age, that had previously been utilised for palatability studies for pet foods.
Sample size:	42 cats: Cats with chronic kidney disease (CKD) (n = 21 cats). This group included: persistently azotaemic cats (n = 15). nonazotaemic cats with abnormal glomerular filtration rate (GFR) measurements (n = 4). nonazotaemic cats with calcium oxalate nephroliths (n = 2). Healthy geriatric cats (n = 21). This group included cats > 10 years of age with the following data collected over a 6-month period: Three normal GFR measurements. Three serum creatinine (sCr) within reference range (0.7–2.1 mg/dL [61.9–185.64 µmol/L]). Three with a urine specific gravity (USG) of >1.040.
Intervention details:	GFR was measured by iohexol clearance tests. GFR results from the healthy geriatric cat population were used to determine median GFR. Impaired GFR was defined as a 30% decrease from the median GFR (< 1.36 ml/min/kg). Cats with GFR < 1.36 mL/min/kg were considered to have CKD and abnormal renal function. CKD cats: sCr: used retrospective data and prospective blood collected. Symmetric dimethylarginine (SDMA): used serum stored in serum banks and prospective blood collected. Measured using liquid chromatography-mass spectroscopy. Range for sCr: 0.7–2.1 mg/dL (61.9–185.64 µmol/L). SDMA > 14 ug/mL was considered cutoff point. Healthy geriatric cats were compared to CKD cats both at the timepoint at which SDMA concentrations were first increased > 14 µg/dL and also when sCr was first increased > 2.1 mg/dL (> 185.64 µmol/L).
Study design:	Retrospective diagnostic test study.
Outcome studied:	Relationship between SDMA and GFR, sCr and GFR, and SDMA and sCr. Sensitivity and specificity of SDMA and sCr for detection of reduced GFR.
Main Findings (relevant to PICO question)	Positive linear relationship between SDMA and sCr (*r *= 0.72). Serum SDMA (*r *=−0.79) and sCr (*r *=* *−0.77) were significantly correlated to GFR (both P < 0.0001). Using SDMA > 14 µg/dL and GFR < 1.36 mL/min/kg as reference intervals SDMA had a 100% sensitivity, 91% specificity, 86%postive predictive value (PPV), 100% negative predictive value (NPV). There were two (2/21) false positive results. Using sCr > 2.1 mg/dL (> 185.64 µmol/L) and GFR < 1.36 ml/min/kgas the upper limit for the reference interval sCr had a 17% sensitivity, 100% specificity, 100% PPV, 70% NPV. In cats with CKD (21/42), serum SDMA concentrations increased above reference interval of 14 µg/dL an average of 14.6 months before sCr concentration increased above the reference interval (RI) of 2.1 mg/dL (185.64 µmol/L). No cats demonstrated elevated creatinine (> 2.1 mg/dL / >185.64 µmol/L) without elevated SDMA (> 14 µg/dL). All healthy geriatric cats (21/42) with normal GFR had SDMA and creatinine within the normal reference interval.
Limitations:	The selection was not randomised in this study, cats were selected based on criteria for the CKD population and healthy geriatric cat population. SDMA concentration was measured on frozen samples, therefore there was no information on the length of time that they had been stored and there is no data available on the stability of SDMA in frozen samples in cats. This study used an upper reference range of 2.1 mg/dL (185.64 µmol/L) for creatinine which is higher than that utilised for International Renal Interest Society (IRIS) staging (Stage 2 CKD is > 140 µmol/L), therefore this may have affected the calculated sensitivity of sCr.

**Hall et al. (2014b):** 

Population:	Healthy cats selected from a colony of 400 domestic shorthairs that had previously been utilised for palatability studies for pet foods.
Sample size:	32 cats.
Intervention details:	Cats were randomised into three study groups and fed either the control diet or either of the experimental diets for a 6-month period The cats were also categorised by age: < 12 years (n = 11) 12–15 years (n = 8) 15 years (n = 10). Blood was collected to measure serum biomarkers of renal function at 1.5, 3 and 6 months. Serum creatinine (sCr) was measured using enzymatic colorimetry, and the reference range of 0.7–2.1 mg/dL (61.88–185.64 µmol/L) was utilised. Symmetric dimethylarginine (SDMA) was measured using liquid chromatography-mass spectroscopy and the upper limit of normal was defined as < 14 µg/dL. Glomerular filtration rate (GFR) was measured by iohexol clearance method (range 1.15–2.73 ml/min/kg).
Study design:	Randomised control trial, cross-sectional study.
Outcome studied:	Effect of diet on body composition, serum biochemistries, renal function markers and GFR (this is not relevant to the PICO question and will not be commented on further in this review). Correlation between: SDMA and GFR. sCr and GFR. Serum biomarkers with age. Serum biomarkers with lean body mass.
Main Findings (relevant to PICO question)	Stronger correlation between SDMA and GFR (*r* = -0.67, P = 0.01) than creatinine and GFR (*r* = -0.44, P = 0.02). sCr was found to be much more affected by age and total lean body mass than SDMA. Three cats were removed due to development of unrelated diseases; therefore 29 cats completed the study.
Limitations:	This study did not specifically look at cats with chronic kidney disease (CKD) and therefore did not specifically look at SDMA and creatinine with reduced GFR. A wider range of GFR analysed would have provided more useful data regarding the correlation. This study used an upper reference range of 2.1 mg/dL (185.64 µmol/L) for creatinine which is higher than that utilised for International Renal Interest Society (IRIS) staging (Stage 2 CKD is > 140 µmol/L), therefore some of the cats classified as ‘healthy’ cats in this study population may have been categorised as having CKD according to the IRIS guidelines.

## Appraisal, application and reflection

Chronic kidney disease (CKD) is a major cause of morbidity in cats, affecting 30–40% of cats over 9 years old, therefore there is an advantage in being able to early identify the condition and monitor renal function for the appropriate application of treatment (Michael et al., 2022). CKD occurs due to the loss of functional renal mass which is most accurately measured using direct measurement of glomerular filtration rate (GFR). GFR is the quantity of glomerular ultrafiltrate formed by the kidneys per unit of time and is measured by plasma or renal clearance of a filtration marker (Finch, 2014). It is directly related to functional renal mass in an adequately hydrated animal (Hall et al., 2016). However, complexity of the test, expense and lack of standardised protocols means that it is not widely utilised in clinical practice. Instead, indirect markers of GFR are more commonly tested (Mack et al., 2021).

The International Renal Interest Society (IRIS) has defined staging of CKD based on the renal biomarkers symmetric dimethylarginine (SDMA) and creatinine to allow for a more practical indirect measure of GFR for clinical practice (IRIS, 2019). SDMA derives from methylation of arginine during proteolysis and more than 90% is excreted via glomerular filtration (Brans et al., 2021; and Braff et al., 2014). Creatinine derives from the dehydration of creatinine phosphate in skeletal muscle and is non protein bound in the circulation, allowing it to be freely filtered by the kidneys (Finch, 2014). SDMA is widely considered a more sensitive biomarker as it has been shown to detect 40% loss of functional renal mass an average of 17 months before elevations in creatinine, whilst creatinine increases at 50–75% loss of functional nephrons (Hall et al., 2014a).

It has been established that creatinine is affected by more non renal factors than SDMA such as lean body mass, age, and diet. However, SDMA and creatinine can also be affected by breed and biological variability and prerenal causes of reduced GFR such as dehydration (Sargent et al., 2021). Furthermore, in the human medical field, SDMA has been found to be affected by various disease states including diabetes, sepsis and thyroid disease in the absence of compromised renal function (Sargent et al., 2021; and Mack et al., 2021). Research within the veterinary field is limited, however in a study of 37 cats by Langhorn et al. (2018) they found SDMA to be significantly lower in cats with diabetes mellitus compared to healthy controls. Additionally, there have been conflicting results regarding the effect of neoplasia on serum SDMA in dogs and cats (Yerramili et al., 2017; Abrams-Ogg et al., 2017; and Coyne et al., 2022).

All four of the studies analysed had clearly described test procedures. The diagnostic test studies analysed have the advantage in that all of the recruited cats have been subjected to the same protocols for data collection as defined by the original prospective studies, therefore minimising the variables. However, Braff et al. (2004) highlighted that the stability of SDMA in frozen samples has not been determined in cats, therefore potentially compromising the validity of these results. The cross-sectional study was part of a randomised, non-blinded control trial for which the population was randomly selected from a colony of cats, therefore reducing selection bias, however, in doing so the study did not examine a wide range of GFR and therefore did not have a representative population for cats with CKD.

Three of the studies (Braff et al., 2014; Hall et al., 2014a; and Brans et al. 2021) found an equal correlation between SDMA and GFR and creatinine and GFR, whilst one showed a stronger correlation between SDMA and GFR (Hall et al., 2014b). Three out of the four studies (Braff et al., 2014; Hall et al., 2014a; and Hall et al. 2014b) used liquid chromatology- mass spectrometry test to measure SDMA, whilst Brans et al. (2021) utilised the IDEXX test, therefore this reduces the comparability of this study. However, they rationalised the use of this test by pointing out that it is both accurate and more applicable to clinical practice. The sample size used by Braff et al. (2014) was also significantly smaller (n = 10) than those used in the other three studies (Brans et al., 2021; Hall et al., 2014a; and Hall et al. 2014b) (n = 32–49), therefore this is a limitation of this study.

The main limitation in comparison of studies looking at the sensitivity and specificity of SDMA and creatinine is the variability of the reference intervals applied for the measurement of serum creatinine (sCr). The IRIS staging guidelines define the reference interval for cats with CKD Stage 2 as between 140–250 µmol/L, however the lower end of this range lies within the normal reference interval for many laboratories (IRIS, 2019). Brans et al. (2021) acknowledged this limitation in their study in which they looked at two different threshold values for creatinine and recognised that with the higher the upper reference limit, the more false negative test results are generated which will ultimately result in a lower sensitivity for creatinine. This is particularly relevant when comparing these studies as Hall et al. (2014a) applied a higher upper reference limit of 185.64 µmol/L for creatinine and documented a lower sensitivity for the detection of impaired GFR compared to SDMA. Similarly, the lower reference range for GFR varied between these studies which may have affected the results; different reference intervals for GFR have been reported and this is partially due to the variability of this measurement due to the effect of age, breed and gender (Finch, 2014).

In conclusion, whilst there is some conflicting evidence, the majority of the studies analysed do not demonstrate that SDMA is superior to creatinine for assessment of GFR and the clinician should be aware of the potential non renal factors that can affect this measurement when interpreting discordant results in practice.

## Methodology

**Search strategy:** 

Databases searched and dates covered:	CAB Abstracts < 1973 to 2023 Week 15 > Medline on OVID Interface (1946–present)
Search strategy:	CAB Abstracts: exp cats (cat or cats or feline*).tw. 1 or 2 glomerular filtration rate (glomerular filtration rate* or gfr).tw. 4 or 5 creatinine creatinine.tw. 7 or 8 (SDMA or Symmetric dimethylarginine).tw. 3 and 6 and 9 and 10 limit 11 to english language Medline: Cats/ (cat or cats or feline*).tw. 1 or 2 Glomerular Filtration Rate/ (glomerular filtration rate* or gfr).tw. 4 or 5 Creatinine creatinine.tw. 7 or 8 (SDMA or Symmetric dimethylarginine).tw. 3 and 6 and 9 and 10 limit 11 to english language The term chronic kidney disease was not included so as to not exclude papers looking at the correlation of the renal biomarkers and glomerular filtration rate in a healthy population.
Dates searches performed:	18 Apr 2023

**Exclusion / inclusion criteria:** 

Exclusion:	Literature examining other species was excluded as this was considered irrelevant to the PICO question. Review papers. Articles not written in the English language. Book chapters. Articles that did not examine the direct association between SDMA, creatinine and GFR.
Inclusion:	Clinical studies. Randomised control trials. Prospective and retrospective cohort studies.

**Search outcome:** 

Database	Number of results	Excluded – Duplicates	Excluded – Not relevant species	Excluded – Did not answer the PICO	Total relevant papers
CAB Abstracts	25	0	4	17	4
Medline	27	0	6	17	4
Total relevant papers when duplicates removed					4

## Acknowledgements

The author would like to thank Emma Place (University Librarian) for assisting with the literature searches.

## ORCiD

Lorna Hardy: https://orcid.org/0000-0002-3452-9469

## Conflict of interest

The author declares no conflicts of interest.

## Disclaimer

Knowledge Summaries are a peer-reviewed article type which aims to answer a clinical question based on the best available current evidence. It does not override the responsibility of the practitioner. Informed decisions should be made by considering such factors as individual clinical expertise and judgement along with patient’s circumstances and owners’ values. Knowledge Summaries are a resource to help inform and any opinions expressed within the Knowledge Summaries are the author’s own and do not necessarily reflect the view of the RCVS Knowledge. Authors are responsible for the accuracy of the content. While the Editor and Publisher believe that all content herein are in accord with current recommendations and practice at the time of publication, they accept no legal responsibility for any errors or omissions, and make no warranty, express or implied, with respect to material contained within. For further information please refer to our Terms of Use.
